# MG53/TRIM72: multi-organ repair protein and beyond

**DOI:** 10.3389/fphys.2024.1377025

**Published:** 2024-04-12

**Authors:** Yong-Fei Wang, Zi-Yi An, Jian-Wen Li, Zi-Kai Dong, Wei-Lin Jin

**Affiliations:** ^1^ The First Clinical Medical College of Lanzhou University, Lanzhou, China; ^2^ Institute of Cancer Neuroscience, Medical Frontier Innovation Research Center, The First Hospital of Lanzhou University, Lanzhou, China

**Keywords:** anti-tumor, cancer cachexia, insulin resistance, MG53, multi-organ protection

## Abstract

MG53, a member of the tripartite motif protein family, possesses multiple functionalities due to its classic membrane repair function, anti-inflammatory ability, and E3 ubiquitin ligase properties. Initially recognized for its crucial role in membrane repair, the therapeutic potential of MG53 has been extensively explored in various diseases including muscle injury, myocardial damage, acute lung injury, and acute kidney injury. However, further research has revealed that the E3 ubiquitin ligase characteristics of MG53 also contribute to the pathogenesis of certain conditions such as diabetic cardiomyopathy, insulin resistance, and metabolic syndrome. Moreover, recent studies have highlighted the anti-tumor effects of MG53 in different types of cancer, such as small cell lung cancer, liver cancer, and colorectal cancer; these effects are closely associated with their E3 ubiquitin ligase activities. In summary, MG53 is a multifunctional protein that participates in important physiological and pathological processes of multiple organs and is a promising therapeutic target for various human diseases. MG53 plays a multi-organ protective role due to its membrane repair function and its exertion of anti-tumor effects due to its E3 ubiquitin ligase properties. In addition, the controversial aspect of MG53’s E3 ubiquitin ligase properties potentially causing insulin resistance and metabolic syndrome necessitates further cross-validation for clarity.

## 1 Introduction

The basic structure of the tripartite motif (TRIM) protein family consists of the N-terminal RING, B-box, and curly helix region TRIM protein (Nisole et al., 2005). Given the high conservation of the triple domain in TRIM proteins, it plays a similar role in cells. The vast majority of TRIM proteins contain RING-finger domains that are primarily involved in ubiquitination in their N-terminal region (Meroni and Diez-Roux, 2005). The binding of the B-box domain to zinc has been shown to play a role in innate immune system defense (Ozato et al., 2008; Esposito et al., 2017). The curly helical domain mediates the interactions between TRIM proteins, allowing proteins to assemble into high molecular weight complexes (Koliopoulos et al., 2016). The variability of the C-terminal domain facilitates the multifunctionality of TRIM proteins, which are pivotal in regulating intracellular signaling and transcription, as well as innate immunity, autophagy, and tumorigenesis (Hatakeyama, 2017).

**FIGURE 1:**
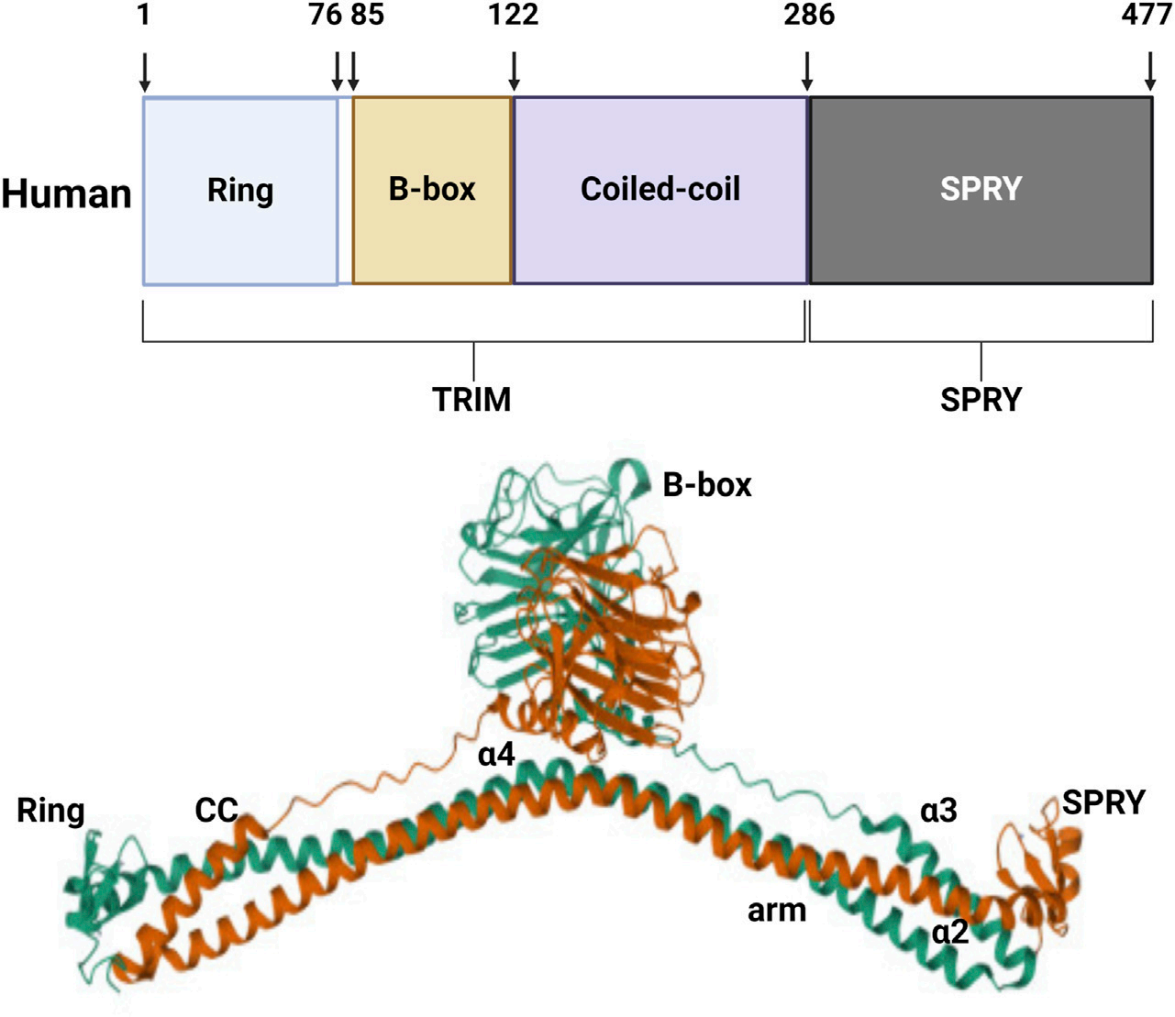
Structure of human MG53 protein. The human MG53 protein consists of the N-terminal RING, B-box, and curly helix regions, as well as the carboxyl terminal SPRY domain.

TRIM protein 72, also known as MG53, was discovered through protein library screening in 2009 (Cai et al., 2009a). The molecular structure of MG53 protein is composed of the N-terminal RING, B-box, and curly helix regions. Except for the carboxyl end which has a SPRY domain, the protein is structurally similar to other members of the TRIM family. The relative molecular weight of the MG53 protein is 53kD, consisting of 477 amino acids (Weisleder et al., 2008) (Figure 1). MG53 is mainly expressed in the heart and skeletal muscle, and RNA analysis has shown that there also exists a small amount of MG53 protein expression in the kidneys, lungs, and cornea (Jia et al., 2014; Duann et al., 2015; Chandler et al., 2019). MG53 is involved in various physiological and pathological processes, including acute membrane repair, intracellular vesicle transport, and skeletal muscle ischemic preconditioning (IPC).

Furthermore, MG53 has been proven a key factor in regulating membrane repair during skeletal muscle injury (Lee et al., 2010).

At present, MG53 has important biological functions, which are crucial for maintaining the normal physiological structure and biological functions of tissues and organs. The therapeutic effect of MG53 depends on its classic membrane repair function and anti-inflammatory ability (Whitson et al., 2021). However, MG53 emerges as a pivotal regulatory factor in the context of impaired glucose metabolism and plays a role in the inhibitory mechanism governing muscle regeneration, potentially impeding treatment outcomes (Wu et al., 2019). Furthermore, recent research has demonstrated the anti-tumor effects of MG53 in various types of cancer including small cell lung cancer, liver cancer, and colorectal cancer. Importantly, these anti-tumor effects are closely related to E3 ubiquitin ligase activities (Du et al., 2023). MG53, akin to a thorny rose, poses a challenging research issue in terms of balancing its relationship and effectively harnessing its therapeutic effects. This review aims to delve into the therapeutic prospects of MG53 while critically examining its positive and negative effects.

## 2 Mechanism of tissue regeneration and repair by MG53

**FIGURE 2:**
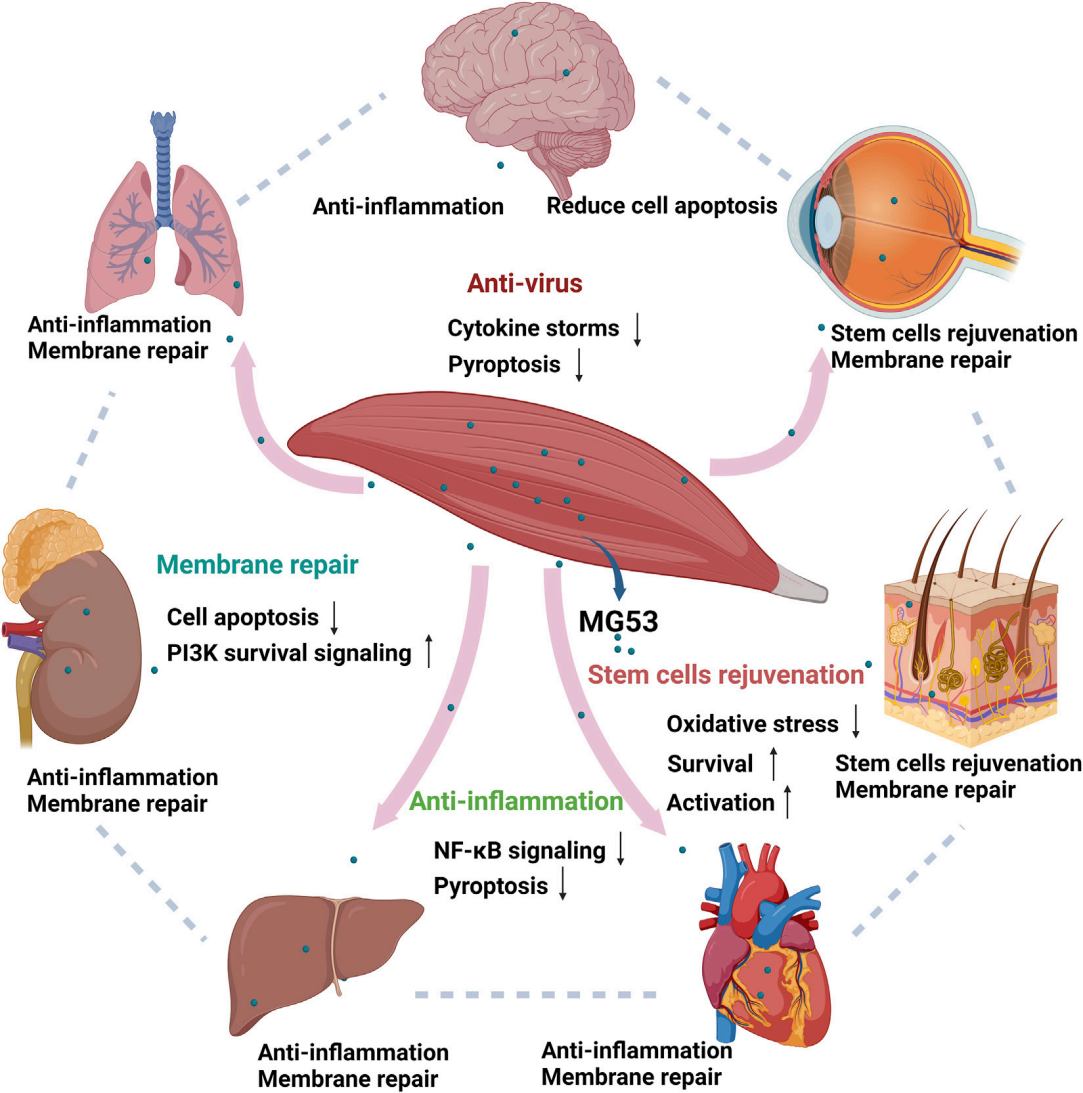
Function of human MG53 protein. The human MG53 protein exhibits four distinct functionalities: membrane repair, anti-inflammatory action, stem cell regeneration and anti-virus effects. The membrane repair function enhances the PI3K survival signaling pathway while inhibiting cellular apoptosis. Its anti-inflammatory capability mitigates NF-κB signaling pathway activation and reduces cell death. Moreover, the regenerative potential of stem cells is activated by MG53 while concurrently suppressing oxidative stress response (Whitson et al., 2021). MG53 also alleviates virus-induced lung injury by alleviating cytokine storms and inhibiting cell pyroptosis. (Created with BioRender.com.)

The tissue repair and regeneration capabilities of MG53 are mediated through four distinct mechanisms, namely, acute cell membrane injury repair, anti-inflammatory function, stem cell rejuvenation, and anti-virus effects (Figure 2). These modes operate independently yet synergistically with one another.

### 2.1 Repair of acute injury to the cell membrane

MG53 exhibits reparative effects on acute cell membrane injuries (McNeil, 2009). Existing studies have summarized the process of membrane repair mediated by MG53 into three main stages. First, the entry of extracellular calcium and oxidants triggers local dynamic responses, followed by the generation of membrane damage signals leading to MG53 oligomerization (Cai et al., 2009a). Subsequently, non-muscle myosin IIA interacts with MG53 and recruits intracellular vesicles containing MG53 to the injury site (Lin et al., 2012). Second, polymerase I and transcription release factor recognize exposed cholesterol at membrane damage sites and bind MG53 with its associated intracellular vesicles to form membrane repair patches (Zhu et al., 2011). Third, upon reaching the membrane, MG53 binds to phosphatidylserine (PS) and serves as a protein scaffold for membrane repair components, such as muscular dystrophy (Hwang et al., 2011; Lek et al., 2013). Finally, the local elevation of Ca2+ promotes the binding of membrane repair patches and plasma membranes, and reseals the membrane to complete the repair (McNeil, 2009). A study demonstrated that recombinant human MG53 (rhMG53) can ameliorate acute lung injury (Jia et al., 2014) while targeting the RISK signal with MG53 enhancing membrane repair and improving ischemic brain injury (Yao et al., 2016). Furthermore, rhMG53 modulates the transforming growth factor beta (TGF-β) signaling pathways to enhance fibrosis remodeling and ultimately facilitates corneal healing (Chandler et al., 2019). In summary, the acute cell membrane repair effect of MG53 is associated with the attenuation of cellular apoptosis and the activation of phosphatidylinositol 3-kinase (PI3K) survival signaling pathway.

### 2.2 Anti-inflammation

The anti-inflammatory function of MG53 is beneficial for tissue damage repair. A study found that knocking down MG53 leads to the activation of nuclear factor-kappa B (NF-κB) signal transduction, which is related to the lysine receptor (Sermersheim et al., 2020). MG53 inhibits toll-like receptor 4/NF-κB pathway and reduces lipopolysaccharide-induced neurotoxicity and neuroinflammation *in vitro* and vivo (Guan et al., 2019a). Moreover, research has demonstrated that MG53 suppresses the NOD-like receptor thermal protein domain associated protein 3/caspase-1/interleukin 1β axis and safeguards human umbilical cord-derived mesenchymal stem cells (hUC-MSCs) against inflammatory damage induced by lipopolysaccharide (Ma et al., 2020). Recent research has indicated that MG53 can alleviate airway inflammation responses in asthmatic mice by regulating the NF-κB pathway (Tan et al., 2023). The anti-inflammatory effect of MG53 is associated with the NF-κB and cell apoptosis signaling pathway.

### 2.3 Stem cell regeneration

The stem cell regenerative capacity of MG53 also plays a pivotal role in organ repair. A recent study demonstrated that the combined therapy involving MG53 and hUC-MSCs effectively mitigates cellular apoptosis while enhancing PI3K/protein kinase B (Akt)/glycogen synthase kinase three beta signal transduction, ultimately promoting neurogenesis (Guan et al., 2019b). MG53 also has a protective effect on bone marrow stem cells (Li et al., 2020). Furthermore, tissue plasminogen activator MG53 mice exhibit remarkable capabilities for dermal wound healing, as well as damage repair and regeneration. Numerous studies have also demonstrated that MG53, present in the bloodstream, functions as a muscle cytokine, which facilitates tissue damage repair and regeneration compared with wild-type mice from the same litter (Bian et al., 2019). The regenerative effect of MG53 on stem cells is related to reducing oxidative stress and increasing stem cell survival.

### 2.4 Anti-virus

A research found that MG53 also has anti-influenza virus infection effects in recent years. An animal experimental study found that rhMG53 can alleviate influenza virus infections in mice. Following mechanism studies have indicated that rhMG53 alleviates virus-induced lung injury by alleviating cytokine storms and inhibiting cell pyroptosis. Moreover, the therapeutic effect of rhMG53 has no effect on viral titer, which is conceptually different from current influenza therapies that directly target viruses (Kenney et al., 2021). Therefore, rhMG53 biological agent can provide a frontline treatment for organ preservation in influenza virus infections and other pathogen-related infections, especially when encountering novel pathogenic viruses such as severe acute respiratory syndrome coronavirus type 2. rhMG53 can also provide a new treatment option (Kenney et al., 2021). Research on the antiviral aspects of MG53 is relatively limited, signaling a novel area of exploration for new functions of MG53 and further research to reveal the relationship between MG53 and viruses.

## 3 Protective effect of MG53 on multiple organs

MG53 has a protective effect on multiple organs, mainly related to tissue repair and regeneration, and its mechanism of action mainly includes acute cell membrane injury repair, anti-inflammation, stem cell rejuvenation, and anti-virus effects.

### 3.1 Protective effect of MG53 on skeletal muscle

MG53 was first reported as a central component in the plasma membrane repair process (McNeil, 2009). When the skeletal muscle cell membranes sustain damage, the intracellular oxidative environment changes, inducing MG53 protein to bind to PS. This phenomenon leads to the recruitment and concentration of MG53 at the site of the muscle fiber rupture, mediating the accumulation of vesicles at the damaged site and promoting the re-closure of the cell membrane. The fusion of vesicles and plasma membranes requires the involvement of calcium, as well as the mediation of caveolin-3 (CaV3). The interaction between MG53 and CaV3 is crucial for cell membrane repair (Cai et al., 2009b; Weisleder et al., 2009; Lek et al., 2013). The newly proposed model for membrane repair involves the formation of cap and shoulder proteins, which are closely associated with MG53 (Demonbreun and McNally, 2016).

The protective efficacy of MG53 against muscle injury has been validated in multiple preclinical animal models, primarily focusing on skeletal muscles. Duchenne muscular dystrophy (DMD) is an X-linked recessive genetic disorder with limited therapeutic options, characterized by muscular structural damage and impaired muscle function. An animal experiment transfected DMD mice with adenovirus expressing human MG53 and revealed the ability of MG53 to mitigate muscle damage in the mouse model through its membrane repair mechanism (Zhu et al., 2015). In addition, amyotrophic lateral sclerosis (ALS) is a fatal neuromuscular disease characterized by progressive loss of motor neuron and muscle atrophy. Studies have shown that systemic administration of rhMG53 protein in ALS mice can protect the diaphragm from injury, maintain the integrity of neuromuscular junctions, and thus slow down the progression of ALS disease (Yi et al., 2021).

### 3.2 Protective effect of MG53 on heart

#### 3.2.1 Ischemia/reperfusion (IR) injury

MG53 can alleviate (I/R) injury. Myocardial infarction, a prevalent cardiac disease, can result in arrhythmia, heart rupture (HF), heart failure and even sudden death in severe cases. Timely restoration of blood perfusion (reperfusion) during myocardial infarction represents the optimal approach to prevent myocardial cell death. However, reperfusion often triggers malignant arrhythmia, leading to secondary damage to the myocardium—known as I/R injury. Currently, the treatment for cardiac I/R injury remains uncertain, with only a limited number of interventions demonstrating efficacy (Kohr et al., 2014). Research has found that MG53 is an important cardio-protective factor involved in the protection against cardiac I/R injury (Liu J. et al., 2015). Similar to endogenous MG53 protein, exogenously administered recombinant MG53 also confers cardioprotection against I/R injury (Zhao et al., 2003). The reduction of cardiac I/R injury in MG53 may be attributed to two mechanisms: 1) Akin to its reparative effect on skeletal muscle cell membranes, MG53 also exhibits a reparative effect on the plasma membrane of cardiac cells (Wang et al., 2010). When myocardial injury occurs, MG53 recruited at the site of injury forms a p85-PI3K/MG53/CaV3 complex with CaV3 and activates the reperfusion injury rescue kinase pathway. 2) The cardioprotective effect of MG53 after IR injury may be related to IPC, which reduces organ damage by briefly blocking blood flow and then restoring perfusion before the pathogenic factor takes effect (Evrengül et al., 2003). IPC is recognized for its ability to avoid severe I/R injury and significantly reduce I/R-induced injury (including reducing myocardial infarction area). Studies have observed that IPC can prevent IR-induced decrease in MG53 expression, and maintaining or enhancing MG53 expression may be one of the cardiac protective mechanisms mediated by IPC (Cao et al., 2010). In addition, recent research has indicated that p55 γ protects the heart against I/R-induced necroptosis by activating the MG53-receptor-interacting protein kinase −3 signaling pathway, a foundational mechanism for IPC-induced cardioprotection (Li Z. et al., 2023).

#### 3.2.2 Cardiac arrhythmias

The normal expression of MG53 is crucial for maintaining cardiac rhythm stability. A study revealed that MG53-mediated membrane transport maintains cell surface K current density to ensure the integrity of myocardial cell action sites (Masumiya et al., 2009). Studies have shown that MG53 expression decreases with myocardial Ito, f—a prominent electrophysiological remodeling issue in myocardial hypertrophy, thereby increasing the susceptibility of the heart to ventricular arrhythmias (Liu et al., 2019). In addition, MG53 may be involved in the occurrence of atrial fibrillation. The common pathogenesis of atrial fibrillation is atrial structural remodeling, and fibrosis is one of the most direct forms of this remodeling process (Sagris et al., 2021). The activation of the TGF- β1/Smad pathway is closely related to the degree of atrial fibrosis and structural remodeling, contributing to the promotion of fibrosis and playing a key role in the formation of atrial fibrillation (Gramley et al., 2010). Research suggests that MG53 may function upstream of the TGF-β1/Smad pathway. MG53 regulates the differentiation of myofibroblasts and promotes cell migration, proliferation, and extracellular matrix synthesis, ultimately leading to atrial fibrosis. In addition, clinical studies have found that MG53 is expressed in the human atrium, and its level increases with the degree of atrial fibrosis, which may lead to atrial fibrillation (Guo et al., 2018).

#### 3.2.3 HF

MG53 can improve HF symptoms. The remodeling of myocardial structure is the main cause of the occurrence and development of HF (Boorsma et al., 2020; Dibb et al., 2022). Researchers have studied the relationship between HF and NF-κB signaling pathway. The NF-κB signaling pathway is an inflammation pathway, and studies have shown that inhibiting NF-κB signaling has a positive effect on HF in mouse models (Poma, 2020). Recent studies have observed the increased activity of NF-κB in HF in humans and mice, whereas the expression of MG53, a negative regulatory factor of NF-κB, is reduced. In addition, long-term treatment with rhMG53 reduces NF-κB activity and improves the contractile function of the mouse heart (Wang et al., 2021). An animal model lacking δ-glycan showed that the overexpression of MG53 can activate cell survival kinases such as Akt, extracellular signal-regulated kinases, and glycogen synthase kinase-3, which improves mouse myocardial contractile function and alleviates HF symptoms in mice (He et al., 2012).

#### 3.2.4 Cardiomyopathy

MG53 can alleviate hypertrophic cardiomyopathy, which is a primary myocardial disease characterized by pathological hypertrophy of the myocardium (Brieler et al., 2017; Teekakirikul et al., 2019). Previous studies have shown that the Akt signaling pathway is associated with myocardial cell hypertrophy (Firth, 2019). A research found that when MG53 is consistently expressed at high levels, the ubiquitination and degradation of insulin substrate receptor 1 (IRS-1) increase, leading to the decrease in mammalian target of rapamycin (mTOR) phosphorylation and the downregulation of Akt signaling, ultimately alleviating myocardial cell hypertrophy (Ham and Mahoney, 2013). Previous studies found that NF-κB signaling pathway is important in cardiac development and is associated with the development of cardiac hypertrophy (Poma, 2020). In addition, studies have shown that MG53 overexpression inhibits NF-κB activity and reduces myocardial hypertrophy (Bryant et al., 2018). Dysfunction of the cardiac transverse (T)-tubule membrane system in the heart is a characteristic of end-stage dilated or ischemic cardiomyopathy, and maintaining normal development and integrity of the T-tubules in the heart is crucial for improving ventricular hypertrophy. Studies have shown that MG53 levels are significantly upregulated in chronic pathological left ventricular pressure overload, which increases membrane vesicle transport and antagonizes T-tubule injury, ultimately inhibiting myocardial structural remodeling. In addition, MG53 deficiency can exacerbate myocardial hypertrophy and dysfunction, which further exacerbates myocardial disease (Xu et al., 2020). MG53 can also alleviate symptoms of septic cardiomyopathy. A study showed that the intravenous injection of rhMG53 can alleviate myocardial damage and improve cardiac function in animal models of infectious cardiomyopathy (Han et al., 2020).

The normal expression of MG53 levels is crucial for maintaining cardiac function. MG53 may become a new protagonist in the precise treatment of cardiomyopathies with the deepening research.

### 3.3 Protective effect on other organs

In addition to the skeletal muscle and heart, MG53 also plays important physiological roles in non-muscular tissues due to its membrane repair function (Figure 2). The overexpression of endogenous MG53 and the exogenous administration of recombinant MG53 protein play an important role in the repair and prevention of acute injury in lungs and kidneys with low MG53 expression (Li H. et al., 2022). In addition, rhMG53 alleviates virus-induced lung injury by alleviating cytokine storms and inhibiting cell pyroptosis (Kenney et al., 2021). In the kidneys of mice with MG53 gene knockout, I/R-induced renal injury is more pronounced, and the intravenous injection of recombinant MG53 protein can alleviate I/R-induced renal injury (Liu et al., 2020). Similar to results in kidneys, current studies have suggested that MG53 can also alleviate I/R-induced lung, brain, and liver injuries in mice (Yao et al., 2016; Yao et al., 2017; Gouchoe et al., 2024). In tissues lacking MG53 expression, the administration of exogenous recombinant MG53 protein has been shown to facilitate brain injury repair (Yao et al., 2016) and enhance wound healing in cases of corneal injury and burns (Wang et al., 2016; Chandler et al., 2019). Moreover, latest research has indicated that MG53 can alleviate skin damage caused by nitrogen mustard (Li H. et al., 2023).

The protective effect of MG53 on multiple organs is attributed to its plasma membrane repair, anti-inflammatory, stem cell regeneration and anti-virus functions (Kenney et al., 2021; Whitson et al., 2021). Hence, MG53 has a potential clinical value in human diseases. However, its application in the treatment of clinical diseases is yet to be realized. Further research is warranted to facilitate the clinical translation of MG53 (Figure 2).

## 4 Anti-tumor effect of MG53

**FIGURE 3:**
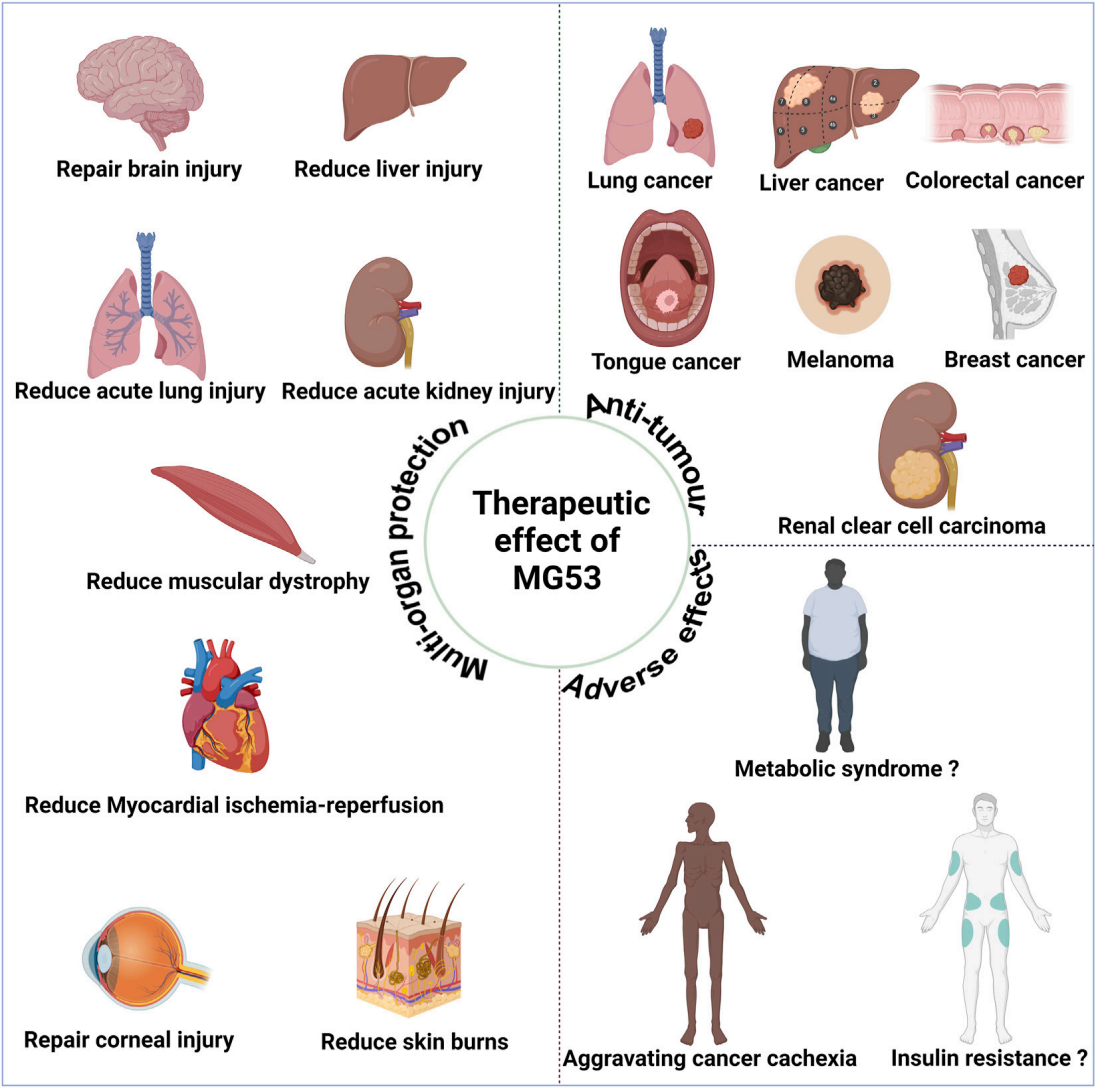
Therapeutic effect of human MG53 protein. MG53 has demonstrated significant therapeutic potential in various diseases due to its membrane repair function, including alleviating muscle malnutrition and facilitating myocardial reperfusion, acute lung injury, acute liver injury, acute kidney injury, brain injury, corneal injury, and skin burn treatment. The E3 ubiquitin ligase properties of MG53 may contribute to exacerbating cancer cachexia and muscle atrophy. Moreover, the controversial aspect of MG53’s E3 ubiquitin ligase properties potentially causing insulin resistance and metabolic syndrome necessitates further cross-validation for clarity. MG53 also exhibits anti-tumor effects in diverse malignancies, such as small cell lung cancer, liver cancer, colorectal cancer, tongue cancer, melanoma, breast cancer, and renal clear cell carcinoma; these anti-tumor effects are closely associated with the E3 ubiquitin ligase activity of MG53. (Created with BioRender.com.)

Since the discovery in 2019 of potential anti-tumor effects associated with MG53 (Chow et al., 2019), MG53 has exhibited anti-tumor effects in various types of tumor, including lung, colon, and liver cancers (Figure 3). Currently, these effects may be attributed to the characteristics of their E3 ubiquitin ligase (Du et al., 2023).

### 4.1 MG53 and lung cancer

The treatment modalities for non-small cell lung cancer (NSCLC) have made significant advancements in the past decade; however, the 5-year survival rate of patients with metastatic non-small cell carcinoma remains considerably low due to multiple mechanisms of drug resistance. Despite the advent of precision medicine offering promising prospects for small cell lung cancer treatment, a pressing need to explore targeted therapeutic approaches remains (Arbour and Riely, 2019). The potential therapeutic application of MG53 in the treatment of NSCLC treatment was initially discovered (Chow et al., 2019). Research findings indicate that MG53 expression is significantly downregulated in metastatic tumors of NSCLC patients compared with non-metastatic tumors, and the knockout of MG53 promotes the growth and metastasis of lung tumors in mice (Chen et al., 2015). Furthermore, a study *in vitro* demonstrated that the administration of rhMG53 protein effectively suppresses stress granule formation and augments the cytotoxicity of cisplatin against human NSCLC cells (Li et al., 2021). The investigation of MG53 for the treatment of NSCLC is still in the preliminary stage and needs further research to facilitate its clinical translation.

### 4.2 MG53 and colorectal cancer

Colorectal cancer ranks the second leading cause of global cancer mortality, projecting an estimated 1.2 million deaths by 2030 (Arnold et al., 2017; Keum and Giovannucci, 2019). Recently conducted studies have demonstrated the anti-colorectal cancer effects of MG53. Investigations have revealed that MG53, functioning as an E3 ubiquitin ligase with the ability to target cyclin D1, hinders the proliferation of colon cancer cells by impeding the cell cycle in the G1 phase and inducing ubiquitin-dependent degradation. Furthermore, when exposed to colorectal cancer carcinogens, MG53 knockout mice exhibit more severe tumor progression compared with their wild-type counterparts (Fang et al., 2023). In a murine tumor xenograft model of multidrug-resistant colorectal cancer, several studies have demonstrated that the combination therapy of doxorubicin and rhMG53 exhibits a significant reduction in tumor size compared with monotherapy with either doxorubicin or rhMG53 (Gupta et al., 2022). The MG53 protein level was found to be significantly lower in colon cancer tissue compared with adjacent tissues in clinical samples of cancer patients. Similarly, decreased levels of the MG53 protein were observed in the serum of patients with colon cancer (Chen et al., 2018). Moreover, low levels of MG53 in tumor tissue were associated with poor prognosis in colon cancer (Fernández-Aceñero et al., 2020). The aforementioned evidence suggests that MG53 exhibits an anti-colorectal cancer effect.

### 4.3 MG53 and liver cancer

The TRIM protein plays a crucial role in the survival, growth, aerobic glycolysis, immune infiltration, and invasion of liver cancer cells (Lu et al., 2022). A study revealed that MG53 exerts inhibitory effects on the malignant progression of hepatocellular carcinoma through its regulation of Ras-related C3 botulinum toxin substrate 1 (RAC1) ubiquitination and degradation while enhancing the chemosensitivity of hepatocellular carcinoma cells to sorafenib treatment by blocking the RAC1/mitogen-activated protein kinase signaling pathway (Ma et al., 2022). However, contrary to findings in patients with colon cancer, a high MG53 expression in patients with hepatocellular carcinoma may be associated with low overall survival rate (Dai et al., 2021). This observation highlights the heterogeneity of MG53 expression across diverse tumor tissues and needs further investigation into the secretion patterns and localization of MG53 during tumorigenesis.

### 4.4 MG53 and other cancers

In addition to exerting anti-tumor effects in lung, colorectal, and liver cancers, studies have demonstrated that MG53 significantly suppresses the proliferation, invasion, and colony formation of tongue cancer cells. Furthermore, the genetic knockout of MG53 accelerates tumor progression in mice (Yin et al., 2019). A study demonstrated that the overexpression of MG53 in melanoma cells confers enhanced resistance to dacarbazine treatment, thereby highlighting its potential as a valuable strategy for improving chemical resistance (Li X. et al., 2022). An analysis of ubiquitin-related genes in the cancer genome atlas queue revealed an association between MG53 and the prognosis of renal clear cell carcinoma (Wu et al., 2021). MG53 can also impede breast cancer progression by suppressing the activation of the PI3K/Akt/mTOR pathway (Wang et al., 2022). Further investigation is warranted to explore the potential anti-tumor effects of MG53 in various malignancies.

Current research strongly suggests that MG53 represents a significant therapeutic target for tumors. However, the precise mechanism underlying its anti-tumor effects remains elusive. Furthermore, the question of whether MG53 is secreted by tumors during their development or if muscle-secreted MG53 circulates to the tumor site poses a challenging issue that has yet to be investigated.

## 5 MG53 promotes the occurrence and development of various diseases

As mentioned above, MG53 has multi-organ protective and anti-tumor effects, but E3 ubiquitin ligase activity determines its adverse effects in the occurrence and development of many diseases. For example, MG53 can negatively regulate muscle synthesis and cause muscle atrophy, as well as lead to diabetes cardiomyopathy, metabolic syndrome, and insulin resistance (Figure 3).

### 5.1 MG53 negatively regulates myogenesis

In cellular contexts, compelling evidence suggests that MG53 functions as a negative regulator of insulin-like growth factor (IGF)-induced myogenesis (Jung and Ko, 2010). MG53, as an E3 ligase, degrades insulin receptors and IRS-1 in a ubiquitin-dependent manner (Song et al., 2013). This capability attenuates the tyrosine phosphorylation of IRS-1 and Akt induced by IGF-1, ultimately exerting a negative regulatory effect on skeletal muscle development (Yi et al., 2013). Findings of previous studies have demonstrated that MG53 overexpression in C2C12 myoblasts exerts an inhibitory effect on the phosphorylation of IRS-1 tyrosine induced by myogenesis and IGF-I, whereas silencing MG53 yields contrasting outcomes (Jung and Ko, 2010). Moreover, MG53 has been shown to induce the ubiquitination and subsequent degradation of focal adhesion kinase (FAK), another kinase, which is believed to exert inhibitory effects on muscle production (Nguyen et al., 2014). This phenomenon pertains to the regulation of heterochromatin remodeling mediated by FAK, the orchestration of myogenin expression during skeletal muscle development, and the modulation of gene expression implicated in membrane fusion (Nguyen et al., 2014).

### 5.2 MG53 can cause diabetes cardiomyopathy

Diabetes cardiomyopathy, characterized by myocardial lipid accumulation, hypertrophy, fibrosis, and cardiac dysfunction, accounts for over 50% of mortality in patients with diabetes (Penpargkul et al., 1981; Trost et al., 2002). It manifests as a unique heart disease with reduced glucose utilization and increased oxidation of free fatty acids in the heart, which leads to lipotoxicity, myocardial cell death, and myocardial fibrosis (Avagimyan et al., 2022). The MG53 overexpression has been demonstrated to induce diabetes cardiomyopathy in mice by activating receptors through the upregulation of peroxisome proliferation α and the inhibition of insulin signal transduction (Liu F. et al., 2015). However, the pathogenesis of diabetes cardiomyopathy remains elusive (Jia et al., 2018).

### 5.3 MG53 can cause metabolic syndrome

The RING domain of MG53 represents a distinctive structural feature of E3 ubiquitin ligase, facilitating the degradation of skeletal muscle IRS-1. In addition, the downregulation of IRS-1 expression can induce systemic insulin resistance (Yi et al., 2013). Metabolic syndrome, characterized by a cluster of disorders including central obesity, dyslipidemia, and hypertension, poses a significant threat to human health as it elevates the risk of cardiovascular disease and type 2 diabetes (Eckel et al., 2005). Given that skeletal muscle accounts for 70%–90% of insulin-mediated glucose processing, the occurrence of metabolic syndrome is closely associated with systemic insulin resistance (Qi et al., 2016). Consequently, skeletal muscle insulin resistance plays a pivotal role in the development of metabolic disorders, and aberrant MG53 expression can contribute to skeletal muscle insulin resistance. Therefore, MG53 assumes a critical function in the pathogenesis of metabolic syndrome. Findings have indicated that the absence of MG53 protein expression does not result in systemic insulin resistance and metabolic disorders, even when a high-fat diet is consumed (Joazeiro and Weissman, 2000). In summary, current research has demonstrated that MG53 mediates the degradation of IRS-1 via ubiquitination, resulting in systemic insulin resistance and ultimately metabolic syndrome. Further research is needed on the relationship between MG53 and insulin resistance.

However, the role of MG53 in insulin signaling and diabetes is controversial. An animal experiment constructed db/db mice with sustained elevation of MG53 in bloodstream. The study found that MG53 did not change the insulin signal transduction and glucose processing in db/db mice. This finding indicates that MG53 may not participate in the development of diabetes (Wang et al., 2020). In addition, a study showed that MG53 is not a key regulatory factor in the insulin signaling pathway in skeletal muscle through vitro and *in vivo* experiments (Philouze et al., 2021). A case-control study of 107 patients with type T2 diabetes and 105 subjects without insulin resistance-related diseases found a lack of association between the levels of MG53 and T2 diabetes (Andaç et al., 2023). Another clinical study found that MG53 could not mark cardiovascular risk and all-cause mortality in patients with type 2 diabetes (Bianchi et al., 2023). The research above shows that MG53 has lacks association with the occurrence of insulin resistance and diabetes. More in-depth research is needed on the relationship between MG53 and negative inotropic effects, diabetes cardiomyopathy, and metabolic syndrome.

## 6 Dialectical analysis of the benefits and drawbacks of MG53 treatment

### 6.1 Optimizing the protective effects of MG53’s multi-organ protective function

MG53 exerts a protective effect on multi-organ by its plasma membrane repair function, encompassing skeletal muscle and myocardial injury, as well as the healing process of acute lung injury, acute kidney injury, corneal damage, and wounds. However, the E3 ubiquitin ligase characteristics of MG53 contribute to the development of various diseases such as diabetes cardiomyopathy and metabolic syndrome, posing significant challenges for the clinical application of MG53. Thus, MG53 can be considered a double-edged sword (Zhang et al., 2016). The optimal utilization of its inherent advantages and the employment of diverse technical approaches to mitigate potential drawbacks are of immense importance. For instance, investigations have successfully attenuated insulin resistance induced by MG53 through targeted disruption of the interaction between MG53 and IRS-1 (Lee et al., 2016). The MG53 mutant currently under construction exhibits the ability to circumvent its E3 ubiquitin ligase activity (Lv et al., 2022), thereby offering a novel therapeutic approach for diabetes, muscle injury diseases, and cardiovascular disorders.

### 6.2 Optimizing the anti-tumor effect of MG53: harnessing E3 ubiquitin ligase activity and monitoring adverse effects

Current research has demonstrated that the anti-tumor efficacy of MG53 is intricately associated with its E3 ubiquitin ligase activity (Du et al., 2023). However, the E3 ubiquitin ligase activity elicits negative inotropic effects, potentially exacerbating cancer cachexia during MG53 treatment, given that a continuous decline in muscle mass represents the primary hallmark of this syndrome (Argilés et al., 2018). Once the tumor progresses to the stage of cancer cachexia, patients’ treatment response and quality of life significantly decline. Therefore, when utilizing MG53 as a therapeutic agent for cancer, it becomes imperative to modify its administration to delay the onset of cancer cachexia as much as possible. Furthermore, given that MG53’s E3 ubiquitin ligase activity can induce insulin resistance, blood glucose levels must be closely monitored during MG53-based cancer treatment.

Future research should focus on elucidating the mechanism of the action of MG53 in tumors, glucose metabolism, and muscle development. On this basis, efforts should be made to preserve the anti-tumor therapeutic ability of MG53 while mitigating its side effects. Currently, MG53 mutants, which retain membrane repair function without compromising glucose metabolism, have been developed by eliminating the E3 ubiquitin ligase properties of MG53 (Lv et al., 2022). However, in the context of cancer treatment, the E3 ubiquitin ligase of MG53 appears to play a pivotal role. Therefore, an imperative challenge for future research lies in harnessing the potent anti-tumor activity of MG53 while mitigating its potential adverse effects. In light of these forthcoming research difficulties, several prospective approaches can be explored: 1) endeavoring to selectively activate MG53 at distinct sites to elicit anti-tumor effects without compromising glucose and muscle metabolism; and 2) delving deeper into the intricate and multifaceted interplay between the E3 ubiquitin ligase of MG53, anti-tumor activity, glucose metabolism, and muscle metabolism. These endeavors hold promise as key avenues toward resolving this conundrum.

## 7 Summary and prospect

Since the initial report on the biological function of MG53, its crucial physiological and pathological roles in skeletal muscle, myocardium, and other various organs have been elucidated, highlighting its potential therapeutic value. Particularly noteworthy is the recent discovery of MG53’s anti-tumor effects across multiple tumor types in 2019. However, although controversies exist, studies have found that MG53 can induce insulin resistance and negative inotropic effects, which require careful consideration. More in-depth research is needed on the mechanism of MG53 in glucose metabolism in the future, and a dialectical view of the advantages and disadvantages of MG53 is crucial for its research and clinical translation.

Furthermore, the current research on the functionality of MG53 predominantly relies on animal models. As an endogenous protein, MG53 exhibits minimal toxicity and elicits negligible immune responses in the body (Weisleder et al., 2012). However, its potential adverse effects pose limitations to clinical trials involving MG53. To overcome these challenges, technological advancements should be employed to mitigate the adverse effects associated with MG53 treatment. In addition, fostering interdisciplinary collaboration between biochemistry and medicine is crucial in facilitating active clinical experimentation. These endeavors will accelerate the development of MG53 protein as a novel therapeutic agent for diverse ailments such as tumors, cardiovascular diseases, and skeletal muscle injuries.

## Abbreviations

AKT, protein kinase B; ALS, amyotrophic lateral sclerosis; CaV3, caveolin-3; DMD, Duchenne muscular dystrophy; FAK, focal adhesion kinase; HF, heart failure; HCM, hypertrophic cardiomyopathy; hUC-MSCs, human umbilical cord-derived mesenchymal stem cells; IPC, ischemic preconditioning; IGF, insulin-like growth factor; IRS-1, insulin substrate receptor 1; I/R, ischemia/reperfusion; MAPK, mitogen-activated protein kinase; mTOR, mammalian target of rapamycin; NF-κB, Nuclear Factor-kappa B; NSCLC, non-small cell lung cancer; PI3K, phosphatidylinositol 3-kinase; RAC1, Ras-related C3 botulinum toxin substrate 1; rhMG53, recombinant human MG53; TGF- β1, transforming growth factor 1; TRIM, Tripartite motif protein.

## References

[B1] Andaç B. Özgün E. Bülbül B. Y. Çolak S. Y. Okur M. Yekdeş A. C. (2023). Association of MG53 with presence of type 2 diabetes mellitus, glycemic control, and diabetic complications. PLoS One 18 (9), e0291333. 10.1371/journal.pone.0291333 37699054 PMC10497120

[B2] Arbour K. C. Riely G. J. (2019). Systemic therapy for locally advanced and metastatic non-small cell lung cancer: a review. Jama 322 (8), 764–774. 10.1001/jama.2019.11058 31454018

[B3] Argilés J. M. Stemmler B. López-Soriano F. J. Busquets S. (2018). Inter-tissue communication in cancer cachexia. Nat. Rev. Endocrinol. 15 (1), 9–20. 10.1038/s41574-018-0123-0 30464312

[B4] Arnold M. Sierra M. S. Laversanne M. Soerjomataram I. Jemal A. Bray F. (2017). Global patterns and trends in colorectal cancer incidence and mortality. Gut 66 (4), 683–691. 10.1136/gutjnl-2015-310912 26818619

[B5] Avagimyan A. Popov S. Shalnova S. (2022). The pathophysiological basis of diabetic cardiomyopathy development. Curr. problems Cardiol. 47 (9), 101156. 10.1016/j.cpcardiol.2022.101156 35192869

[B6] Bian Z. Wang Q. Zhou X. Tan T. Park K. H. Kramer H. F. (2019). Sustained elevation of MG53 in the bloodstream increases tissue regenerative capacity without compromising metabolic function. Nat. Commun. 10 (1), 4659. 10.1038/s41467-019-12483-0 31604915 PMC6789113

[B7] Bianchi C. Vaccaro O. Distaso M. Franzini L. Raggi F. Solini A. (2023). MG53 does not mark cardiovascular risk and all-cause mortality in subjects with type 2 diabetes: a prospective, observational study. Diabetes Res. Clin. Pract. 204, 110916. 10.1016/j.diabres.2023.110916 37748712

[B8] Boorsma E. M. Ter Maaten J. M. Damman K. Dinh W. Gustafsson F. Goldsmith S. (2020). Congestion in heart failure: a contemporary look at physiology, diagnosis and treatment. Nat. Rev. Cardiol. 17 (10), 641–655. 10.1038/s41569-020-0379-7 32415147

[B9] Brieler J. Breeden M. A. Tucker J. (2017). Cardiomyopathy: an overview. Am. Fam. physician 96 (10), 640–646. 29431384

[B10] Bryant S. M. Kong C. H. T. Watson J. J. Gadeberg H. C. Roth D. M. Patel H. H. (2018). Caveolin-3 KO disrupts t-tubule structure and decreases t-tubular I(Ca) density in mouse ventricular myocytes. Am. J. Physiol. Heart Circ. Physiol. 315 (5), H1101–h1111. 10.1152/ajpheart.00209.2018 30028203 PMC6415741

[B11] Cai C. Masumiya H. Weisleder N. Matsuda N. Nishi M. Hwang M. (2009a). MG53 nucleates assembly of cell membrane repair machinery. Nat. Cell Biol. 11 (1), 56–64. 10.1038/ncb1812 19043407 PMC2990407

[B12] Cai C. Masumiya H. Weisleder N. Pan Z. Nishi M. Komazaki S. (2009b). MG53 regulates membrane budding and exocytosis in muscle cells. J. Biol. Chem. 284 (5), 3314–3322. 10.1074/jbc.M808866200 19029292 PMC2631961

[B13] Cao C. M. Zhang Y. Weisleder N. Ferrante C. Wang X. Lv F. (2010). MG53 constitutes a primary determinant of cardiac ischemic preconditioning. Circulation 121 (23), 2565–2574. 10.1161/circulationaha.110.954628 20516375

[B14] Chandler H. L. Tan T. Yang C. Gemensky-Metzler A. J. Wehrman R. F. Jiang Q. (2019). MG53 promotes corneal wound healing and mitigates fibrotic remodeling in rodents. Commun. Biol. 2, 71. 10.1038/s42003-019-0316-7 30793049 PMC6382791

[B15] Chen S. Sanjana N. E. Zheng K. Shalem O. Lee K. Shi X. (2015). Genome-wide CRISPR screen in a mouse model of tumor growth and metastasis. Cell 160 (6), 1246–1260. 10.1016/j.cell.2015.02.038 25748654 PMC4380877

[B16] Chen Z. Yin X. Li K. Chen S. Li H. Li Y. (2018). Serum levels of TRIM72 are lower among patients with colon cancer: identification of a potential diagnostic marker. Tohoku J. Exp. Med. 245 (1), 61–68. 10.1620/tjem.245.61 29806630

[B17] Chow R. D. Wang G. Ye L. Codina A. Kim H. R. Shen L. (2019). *In vivo* profiling of metastatic double knockouts through CRISPR-Cpf1 screens. Nat. methods 16 (5), 405–408. 10.1038/s41592-019-0371-5 30962622 PMC6592845

[B18] Dai W. Wang J. Wang Z. Xiao Y. Li J. Hong L. (2021). Comprehensive analysis of the prognostic values of the TRIM family in hepatocellular carcinoma. Front. Oncol. 11, 767644. 10.3389/fonc.2021.767644 35004288 PMC8733586

[B19] Demonbreun A. R. McNally E. M. (2016). Plasma membrane repair in health and disease. Curr. Top. Membr. 77, 67–96. 10.1016/bs.ctm.2015.10.006 26781830 PMC4827257

[B20] Dibb K. M. Louch W. E. Trafford A. W. (2022). Cardiac transverse tubules in physiology and heart failure. Annu. Rev. physiology 84, 229–255. 10.1146/annurev-physiol-061121-040148 34780259

[B21] Du Y. Li T. Yi M. (2023). Is MG53 a potential therapeutic target for cancer? Front. Endocrinol. 14, 1295349. 10.3389/fendo.2023.1295349 PMC10684902 38033997

[B22] Duann P. Li H. Lin P. Tan T. Wang Z. Chen K. (2015). MG53-mediated cell membrane repair protects against acute kidney injury. Sci. Transl. Med. 7 (279), 279ra36. 10.1126/scitranslmed.3010755 PMC4524523 25787762

[B23] Eckel R. H. Grundy S. M. Zimmet P. Z. (2005). The metabolic syndrome. Lancet London, Engl. 365 (9468), 1415–1428. 10.1016/s0140-6736(05)66378-7 15836891

[B24] Esposito D. Koliopoulos M. G. Rittinger K. (2017). Structural determinants of TRIM protein function. Biochem. Soc. Trans. 45 (1), 183–191. 10.1042/bst20160325 28202672

[B25] Evrengül H. Dursunoğlu D. Semiz E. (2003). Ischemic preconditioning. Anadolu kardiyoloji dergisi AKD = Anatol. J. Cardiol. 3 (2), 144–149. 12826510

[B26] Fang M. Wu H. K. Pei Y. Zhang Y. Gao X. He Y. (2023). E3 ligase MG53 suppresses tumor growth by degrading cyclin D1. Signal Transduct. Target. Ther. 8 (1), 263. 10.1038/s41392-023-01458-9 37414783 PMC10326024

[B27] Fernández-Aceñero M. J. Cruz M. Sastre-Varela J. Casal J. I. Nieto M. A. C. Del Puerto-Nevado L. (2020). TRIM72 immunohistochemical expression can predict relapse in colorectal carcinoma. Pathology Oncol. Res. POR 26 (2), 861–865. 10.1007/s12253-019-00629-w 30852740

[B28] Firth J. (2019). Cardiology: hypertrophic cardiomyopathy. Clin. Med. Lond. Engl. 19 (1), 61–63. 10.7861/clinmedicine.19-1-61 PMC6399630 30651247

[B29] Gouchoe D. A. Lee Y. G. Kim J. L. Zhang Z. Marshall J. M. Ganapathi A. (2024). Mitsugumin 53 mitigation of ischemia-reperfusion injury in a mouse model. J. Thorac. Cardiovasc Surg. 167 (3), e48–e58. 10.1016/j.jtcvs.2023.08.005 37562677 PMC12047617

[B30] Gramley F. Lorenzen J. Koellensperger E. Kettering K. Weiss C. Munzel T. (2010). Atrial fibrosis and atrial fibrillation: the role of the TGF-β1 signaling pathway. Int. J. Cardiol. 143 (3), 405–413. 10.1016/j.ijcard.2009.03.110 19394095

[B31] Guan F. Huang T. Wang X. Xing Q. Gumpper K. Li P. (2019b). The TRIM protein Mitsugumin 53 enhances survival and therapeutic efficacy of stem cells in murine traumatic brain injury. Stem Cell Res. Ther. 10 (1), 352. 10.1186/s13287-019-1433-4 31779687 PMC6883632

[B32] Guan F. Zhou X. Li P. Wang Y. Liu M. Li F. (2019a). MG53 attenuates lipopolysaccharide-induced neurotoxicity and neuroinflammation via inhibiting TLR4/NF-κB pathway *in vitro* and *in vivo* . Prog. neuro-psychopharmacology Biol. psychiatry 95, 109684. 10.1016/j.pnpbp.2019.109684 PMC6708450 31260721

[B33] Guo J. Jia F. Jiang Y. Li Q. Yang Y. Xiao M. (2018). Potential role of MG53 in the regulation of transforming-growth-factor-β1-induced atrial fibrosis and vulnerability to atrial fibrillation. Exp. Cell Res. 362 (2), 436–443. 10.1016/j.yexcr.2017.12.007 29233682

[B34] Gupta P. Li H. Zhang G. N. Barbuti A. M. Yang Y. Lin P. H. (2022). MG53 inhibits cellular proliferation and tumor progression in colorectal carcinoma. Int. J. Biol. Sci. 18 (14), 5221–5229. 10.7150/ijbs.67869 36147477 PMC9461659

[B35] Ham Y. M. Mahoney S. J. (2013). Compensation of the AKT signaling by ERK signaling in transgenic mice hearts overexpressing TRIM72. Exp. Cell Res. 319 (10), 1451–1462. 10.1016/j.yexcr.2013.02.016 23567182

[B36] Han X. Chen D. Liufu N. Ji F. Zeng Q. Yao W. (2020). MG53 protects against sepsis-induced myocardial dysfunction by upregulating peroxisome proliferator-activated receptor-α. Oxid. Med. Cell Longev. 2020, 7413693. 10.1155/2020/7413693 32908637 PMC7474382

[B37] Hatakeyama S. (2017). TRIM family proteins: roles in autophagy, immunity, and carcinogenesis. Trends Biochem. Sci. 42 (4), 297–311. 10.1016/j.tibs.2017.01.002 28118948

[B38] He B. Tang R. H. Weisleder N. Xiao B. Yuan Z. Cai C. (2012). Enhancing muscle membrane repair by gene delivery of MG53 ameliorates muscular dystrophy and heart failure in δ-Sarcoglycan-deficient hamsters. Mol. Ther. J. Am. Soc. Gene Ther. 20 (4), 727–735. 10.1038/mt.2012.5 PMC3321592 22314291

[B39] Hwang M. Ko J. K. Weisleder N. Takeshima H. Ma J. (2011). Redox-dependent oligomerization through a leucine zipper motif is essential for MG53-mediated cell membrane repair. Am. J. Physiol. Cell Physiol. 301 (1), C106–C114. 10.1152/ajpcell.00382.2010 21525429 PMC3129825

[B40] Jia G. Hill M. A. Sowers J. R. (2018). Diabetic cardiomyopathy: an update of mechanisms contributing to this clinical entity. Circulation Res. 122 (4), 624–638. 10.1161/circresaha.117.311586 29449364 PMC5819359

[B41] Jia Y. Chen K. Lin P. Lieber G. Nishi M. Yan R. (2014). Treatment of acute lung injury by targeting MG53-mediated cell membrane repair. Nat. Commun. 5, 4387. 10.1038/ncomms5387 25034454 PMC4109002

[B42] Joazeiro C. A. Weissman A. M. (2000). RING finger proteins: mediators of ubiquitin ligase activity. Cell 102 (5), 549–552. 10.1016/s0092-8674(00)00077-5 11007473

[B43] Jung S. Y. Ko Y. G. (2010). TRIM72, a novel negative feedback regulator of myogenesis, is transcriptionally activated by the synergism of MyoD (or myogenin) and MEF2. Biochem. Biophys. Res. Commun. 396 (2), 238–245. 10.1016/j.bbrc.2010.04.072 20399744

[B44] Kenney A. D. Li Z. Bian Z. Zhou X. Whitson B. A. Tan T. (2021). Recombinant MG53 protein protects mice from lethal influenza virus infection. Am. J. Respir. Crit. care Med. 203 (2), 254–257. 10.1164/rccm.202007-2908LE 33031705 PMC7874416

[B45] Keum N. Giovannucci E. (2019). Global burden of colorectal cancer: emerging trends, risk factors and prevention strategies. Nat. Rev. Gastroenterology hepatology 16 (12), 713–732. 10.1038/s41575-019-0189-8 31455888

[B46] Kohr M. J. Evangelista A. M. Ferlito M. Steenbergen C. Murphy E. (2014). S-nitrosylation of TRIM72 at cysteine 144 is critical for protection against oxidation-induced protein degradation and cell death. J. Mol. Cell. Cardiol. 69, 67–74. 10.1016/j.yjmcc.2014.01.010 24487118 PMC3954155

[B47] Koliopoulos M. G. Esposito D. Christodoulou E. Taylor I. A. Rittinger K. (2016). Functional role of TRIM E3 ligase oligomerization and regulation of catalytic activity. EMBO J. 35 (11), 1204–1218. 10.15252/embj.201593741 27154206 PMC4864278

[B48] Lee C. S. Yi J. S. Jung S. Y. Kim B. W. Lee N. R. Choo H. J. (2010). TRIM72 negatively regulates myogenesis via targeting insulin receptor substrate-1. Cell death Differ. 17 (8), 1254–1265. 10.1038/cdd.2010.1 20139895

[B49] Lee H. Park J. J. Nguyen N. Hong J. Kim S. H. Song W. Y. (2016). MG53-IRS-1 (mitsugumin 53-insulin receptor substrate-1) interaction disruptor sensitizes insulin signaling in skeletal muscle. J. Biol. Chem. 291 (52), 26627–26635. 10.1074/jbc.M116.754424 27810898 PMC5207173

[B50] Lek A. Evesson F. J. Lemckert F. A. Redpath G. M. I. Lueders A. K. Turnbull L. (2013). Calpains, cleaved mini-dysferlinC72, and L-type channels underpin calcium-dependent muscle membrane repair. J. Neurosci. official J. Soc. Neurosci. 33 (12), 5085–5094. 10.1523/jneurosci.3560-12.2013 PMC6704986 23516275

[B51] Li H. Li Z. Li X. Cai C. Zhao S. L. Merritt R. E. (2023b). MG53 mitigates nitrogen mustard-induced skin injury. Cells 12 (14), 1915. 10.3390/cells12141915 37508578 PMC10378386

[B52] Li H. Lin P. H. Gupta P. Li X. Zhao S. L. Zhou X. (2021). MG53 suppresses tumor progression and stress granule formation by modulating G3BP2 activity in non-small cell lung cancer. Mol. cancer 20 (1), 118. 10.1186/s12943-021-01418-3 34521423 PMC8439062

[B53] Li H. Rosas L. Li Z. Bian Z. Li X. Choi K. (2022a). MG53 attenuates nitrogen mustard-induced acute lung injury. J. Cell Mol. Med. 26 (7), 1886–1895. 10.1111/jcmm.16917 35199443 PMC8980905

[B54] Li X. Jiang M. Tan T. Narasimhulu C. A. Xiao Y. Hao H. (2020). N-acetylcysteine prevents oxidized low-density lipoprotein-induced reduction of MG53 and enhances MG53 protective effect on bone marrow stem cells. J. Cell Mol. Med. 24 (1), 886–898. 10.1111/jcmm.14798 31742908 PMC6933383

[B55] Li X. Yang C. Luo N. Yang Y. Guo Y. Chen P. (2022b). Ubiquitination and degradation of MGMT by TRIM72 increases the sensitivity of uveal melanoma cells to Dacarbazine treatment. Cancer biomarkers Sect. A Dis. markers 34 (2), 275–284. 10.3233/cbm-210345 PMC12364275 34958003

[B56] Li Z. Dai R. Chen M. Huang L. Zhu K. Li M. (2023a). p55γ degrades RIP3 via MG53 to suppress ischaemia-induced myocardial necroptosis and mediates cardioprotection of preconditioning. Cardiovasc Res. 119 (14), 2421–2440. 10.1093/cvr/cvad123 37527538

[B57] Lin P. Zhu H. Cai C. Wang X. Cao C. Xiao R. (2012). Nonmuscle myosin IIA facilitates vesicle trafficking for MG53-mediated cell membrane repair. Faseb J. 26 (5), 1875–1883. 10.1096/fj.11-188599 22253476 PMC3336789

[B58] Liu C. Hu Y. H. Han Y. Wang Y. B. Zhang Y. Zhang X. Q. (2020). MG53 protects against contrast-induced acute kidney injury by reducing cell membrane damage and apoptosis. Acta Pharmacol. Sin. 41 (11), 1457–1464. 10.1038/s41401-020-0420-8 32424239 PMC7656601

[B59] Liu F. Song R. Feng Y. Guo J. Chen Y. Zhang Y. (2015b). Upregulation of MG53 induces diabetic cardiomyopathy through transcriptional activation of peroxisome proliferation-activated receptor α. Circulation 131 (9), 795–804. 10.1161/circulationaha.114.012285 25637627

[B60] Liu J. Zhu H. Zheng Y. Xu Z. Li L. Tan T. (2015a). Cardioprotection of recombinant human MG53 protein in a porcine model of ischemia and reperfusion injury. J. Mol. Cell. Cardiol. 80, 10–19. 10.1016/j.yjmcc.2014.12.010 25533937 PMC4512204

[B61] Liu W. Wang G. Zhang C. Ding W. Cheng W. Luo Y. (2019). MG53, A novel regulator of KChIP2 and Ito,f, plays a critical role in electrophysiological remodeling in cardiac hypertrophyf, plays a critical role in electrophysiological remodeling in cardiac hypertrophy. Circulation 139 (18), 2142–2156. 10.1161/circulationaha.118.029413 30760025

[B62] Lu K. Pan Y. Huang Z. Liang H. Ding Z. Y. Zhang B. (2022). TRIM proteins in hepatocellular carcinoma. J. Biomed. Sci. 29 (1), 69. 10.1186/s12929-022-00854-7 36100865 PMC9469581

[B63] Lv F. Wang Y. Shan D. Guo S. Chen G. Jin L. (2022). Blocking MG53(S255) phosphorylation protects diabetic heart from ischemic injury. Circulation Res. 131 (12), 962–976. 10.1161/circresaha.122.321055 36337049 PMC9770150

[B64] Ma S. Wang Y. Zhou X. Li Z. Zhang Z. Wang Y. (2020). MG53 protects hUC-MSCs against inflammatory damage and synergistically enhances their efficacy in neuroinflammation injured brain through inhibiting NLRP3/caspase-1/IL-1β Axis. ACS Chem. Neurosci. 11 (17), 2590–2601. 10.1021/acschemneuro.0c00268 32786312

[B65] Ma X. Ma X. Zhu L. Zhao Y. Chen M. Li T. (2022). The E3 ubiquitin ligase MG53 inhibits hepatocellular carcinoma by targeting RAC1 signaling. Oncogenesis 11 (1), 40. 10.1038/s41389-022-00414-6 35858925 PMC9300626

[B66] Masumiya H. Asaumi Y. Nishi M. Minamisawa S. Adachi-Akahane S. Yoshida M. (2009). Mitsugumin 53-mediated maintenance of K+ currents in cardiac myocytes. Channels (Austin, Tex.) 3 (1), 6–11. 10.4161/chan.3.1.7571 19202355 PMC3013504

[B67] McNeil P. (2009). Membrane repair redux: redox of MG53. Nat. Cell Biol. 11 (1), 7–9. 10.1038/ncb0109-7 19122591

[B68] Meroni G. Diez-Roux G. (2005). TRIM/RBCC, a novel class of 'single protein RING finger' E3 ubiquitin ligases. BioEssays news Rev. Mol. Cell. Dev. Biol. 27 (11), 1147–1157. 10.1002/bies.20304 16237670

[B69] Nguyen N. Yi J. S. Park H. Lee J. S. Ko Y. G. (2014). Mitsugumin 53 (MG53) ligase ubiquitinates focal adhesion kinase during skeletal myogenesis. J. Biol. Chem. 289 (6), 3209–3216. 10.1074/jbc.M113.525154 24344130 PMC3916525

[B70] Nisole S. Stoye J. P. Saïb A. (2005). TRIM family proteins: retroviral restriction and antiviral defence. Nat. Rev. Microbiol. 3 (10), 799–808. 10.1038/nrmicro1248 16175175

[B71] Ozato K. Shin D. M. Chang T. H. Morse H. C. 3rd (2008). TRIM family proteins and their emerging roles in innate immunity. Nat. Rev. Immunol. 8 (11), 849–860. 10.1038/nri2413 18836477 PMC3433745

[B72] Penpargkul S. Fein F. Sonnenblick E. H. Scheuer J. (1981). Depressed cardiac sarcoplasmic reticular function from diabetic rats. J. Mol. Cell. Cardiol. 13 (3), 303–309. 10.1016/0022-2828(81)90318-7 6115064

[B73] Philouze C. Turban S. Cremers B. Caliez A. Lamarche G. Bernard C. (2021). MG53 is not a critical regulator of insulin signaling pathway in skeletal muscle. PLoS One 16 (2), e0245179. 10.1371/journal.pone.0245179 33566837 PMC7875368

[B74] Poma P. (2020). NF-κB and disease. Int. J. Mol. Sci. 21 (23), 9181. 10.3390/ijms21239181 33276434 PMC7730361

[B75] Qi J. Yang B. Ren C. Fu J. Zhang J. (2016). Swimming exercise alleviated insulin resistance by regulating tripartite motif family protein 72 expression and AKT signal pathway in sprague-dawley rats fed with high-fat diet. J. diabetes Res. 2016, 1564386. 10.1155/2016/1564386 27843952 PMC5098085

[B76] Sagris M. Vardas E. P. Theofilis P. Antonopoulos A. S. Oikonomou E. Tousoulis D. (2021). Atrial fibrillation: pathogenesis, predisposing factors, and genetics. Int. J. Mol. Sci. 23 (1), 6. 10.3390/ijms23010006 35008432 PMC8744894

[B77] Sermersheim M. Kenney A. D. Lin P. H. McMichael T. M. Cai C. Gumpper K. (2020). MG53 suppresses interferon-β and inflammation via regulation of ryanodine receptor-mediated intracellular calcium signaling. Nat. Commun. 11 (1), 3624. 10.1038/s41467-020-17177-6 32681036 PMC7368064

[B78] Song R. Peng W. Zhang Y. Lv F. Wu H. K. Guo J. (2013). Central role of E3 ubiquitin ligase MG53 in insulin resistance and metabolic disorders. Nature 494 (7437), 375–379. 10.1038/nature11834 23354051

[B79] Tan S. Li M. Song X. (2023). MG53 alleviates airway inflammatory responses by regulating nuclear factor-κB pathway in asthmatic mice. Allergol. Immunopathol. Madr. 51 (4), 175–181. 10.15586/aei.v51i4.880 37422795

[B80] Teekakirikul P. Zhu W. Huang H. C. Fung E. (2019). Hypertrophic cardiomyopathy: an overview of genetics and management. Biomolecules 9 (12), 878. 10.3390/biom9120878 31888115 PMC6995589

[B81] Trost S. U. Belke D. D. Bluhm W. F. Meyer M. Swanson E. Dillmann W. H. (2002). Overexpression of the sarcoplasmic reticulum Ca(2+)-ATPase improves myocardial contractility in diabetic cardiomyopathy. Diabetes 51 (4), 1166–1171. 10.2337/diabetes.51.4.1166 11916940

[B82] Wang C. Wang H. Wu D. Hu J. Wu W. Zhang Y. (2016). A novel perspective for burn-induced myopathy: membrane repair defect. Sci. Rep. 6, 31409. 10.1038/srep31409 27545095 PMC4992861

[B83] Wang Q. Bian Z. Jiang Q. Wang X. Zhou X. Park K. H. (2020). MG53 does not manifest the development of diabetes in db/db mice. Diabetes 69 (5), 1052–1064. 10.2337/db19-0807 32139593 PMC7171965

[B84] Wang X. Li X. Ong H. Tan T. Park K. H. Bian Z. (2021). MG53 suppresses NF-κB activation to mitigate age-related heart failure. JCI Insight 6 (17), e148375. 10.1172/jci.insight.148375 34292883 PMC8492351

[B85] Wang X. Xie W. Zhang Y. Lin P. Han L. Han P. (2010). Cardioprotection of ischemia/reperfusion injury by cholesterol-dependent MG53-mediated membrane repair. Circulation Res. 107 (1), 76–83. 10.1161/circresaha.109.215822 20466981

[B86] Wang Z. Li H. Wang H. Li X. Zhang Q. Wang H. (2022). TRIM72 exerts antitumor effects in breast cancer and modulates lactate production and MCT4 promoter activity by interacting with PPP3CA. Anti-cancer drugs 33 (5), 489–501. 10.1097/cad.0000000000001304 35324524 PMC8997701

[B87] Weisleder N. Takeshima H. Ma J. (2008). Immuno-proteomic approach to excitation-contraction coupling in skeletal and cardiac muscle: molecular insights revealed by the mitsugumins. Cell calcium 43 (1), 1–8. 10.1016/j.ceca.2007.10.006 18061662 PMC3059838

[B88] Weisleder N. Takeshima H. Ma J. (2009). Mitsugumin 53 (MG53) facilitates vesicle trafficking in striated muscle to contribute to cell membrane repair. Commun. Integr. Biol. 2 (3), 225–226. 10.4161/cib.2.3.8077 19641737 PMC2717527

[B89] Weisleder N. Takizawa N. Lin P. Wang X. Cao C. Zhang Y. (2012). Recombinant MG53 protein modulates therapeutic cell membrane repair in treatment of muscular dystrophy. Sci. Transl. Med. 4 (139), 139ra85. 10.1126/scitranslmed.3003921 PMC3777623 22723464

[B90] Whitson B. A. Tan T. Gong N. Zhu H. Ma J. (2021). Muscle multiorgan crosstalk with MG53 as a myokine for tissue repair and regeneration. Curr. Opin. Pharmacol. 59, 26–32. 10.1016/j.coph.2021.04.005 34052525 PMC8513491

[B91] Wu H. K. Zhang Y. Cao C. M. Hu X. Fang M. Yao Y. (2019). Glucose-sensitive myokine/cardiokine MG53 regulates systemic insulin response and metabolic homeostasis. Circulation 139 (7), 901–914. 10.1161/circulationaha.118.037216 30586741

[B92] Wu Y. Zhang X. Wei X. Feng H. Hu B. Deng Z. (2021). Development of an individualized ubiquitin prognostic signature for clear cell renal cell carcinoma. Front. Cell Dev. Biol. 9, 684643. 10.3389/fcell.2021.684643 34239875 PMC8258262

[B93] Xu L. Wang H. Jiang F. Sun H. Zhang D. (2020). LncRNA AK045171 protects the heart from cardiac hypertrophy by regulating the SP1/MG53 signalling pathway. Aging 12 (4), 3126–3139. 10.18632/aging.102668 32087602 PMC7066930

[B94] Yao W. Li H. Han X. Chen C. Zhang Y. Tai W. L. (2017). MG53 anchored by dysferlin to cell membrane reduces hepatocyte apoptosis which induced by ischaemia/reperfusion injury *in vivo* and *in vitro* . J. Cell Mol. Med. 21 (10), 2503–2513. 10.1111/jcmm.13171 28401647 PMC5618678

[B95] Yao Y. Zhang B. Zhu H. Li H. Han Y. Chen K. (2016). MG53 permeates through blood-brain barrier to protect ischemic brain injury. Oncotarget 7 (16), 22474–22485. 10.18632/oncotarget.7965 26967557 PMC5008374

[B96] Yi J. Li A. Li X. Park K. Zhou X. Yi F. (2021). MG53 preserves neuromuscular junction integrity and alleviates ALS disease progression. Antioxidants (Basel) 10 (10), 1522. 10.3390/antiox10101522 34679657 PMC8532806

[B97] Yi J. S. Park J. S. Ham Y. M. Nguyen N. Lee N. R. Hong J. (2013). MG53-induced IRS-1 ubiquitination negatively regulates skeletal myogenesis and insulin signalling. Nat. Commun. 4, 2354. 10.1038/ncomms3354 23965929 PMC3941707

[B98] Yin W. Liu Y. Bian Z. (2019). MG53 inhibits the progression of tongue cancer cells through regulating PI3K-AKT signaling pathway: evidence from 3D cell culture and animal model. Small 15 (8), e1805492. 10.1002/smll.201805492 30690890

[B99] Zhang Y. Wu H. K. Lv F. X. Xiao R. P. (2016). MG53 is a double-edged sword for human diseases. Sheng li xue bao Acta Physiol. Sin. 68 (4), 505–516. 27546510

[B100] Zhao Z. Q. Corvera J. S. Halkos M. E. Kerendi F. Wang N. P. Guyton R. A. (2003). Inhibition of myocardial injury by ischemic postconditioning during reperfusion: comparison with ischemic preconditioning. Am. J. Physiol. Heart Circ. Physiol. 285 (2), H579–H588. 10.1152/ajpheart.01064.2002 12860564

[B101] Zhu H. Hou J. Roe J. L. Park K. H. Tan T. Zheng Y. (2015). Amelioration of ischemia-reperfusion-induced muscle injury by the recombinant human MG53 protein. Muscle Nerve 52 (5), 852–858. 10.1002/mus.24619 25703692 PMC4545465

[B102] Zhu H. Lin P. De G. Choi K. h. Takeshima H. Weisleder N. (2011). Polymerase transcriptase release factor (PTRF) anchors MG53 protein to cell injury site for initiation of membrane repair. J. Biol. Chem. 286 (15), 12820–12824. 10.1074/jbc.C111.221440 21343302 PMC3075629

